# Idiopathic hypereosinophilic syndrome presenting as capsular warning syndrome: A case report and literature review

**DOI:** 10.1097/MD.0000000000034682

**Published:** 2023-09-08

**Authors:** Ze-Hua Lai, Kai-Qi Ding, Xuan-Qiang Tu, Yuan-Yue Song, Li-Li Zeng

**Affiliations:** a Department of Neurology and Institute of Neurology, Ruijin Hospital, Shanghai Jiao Tong University School of Medicine, Shanghai, China.

**Keywords:** capsular warning syndrome, case report, idiopathic hypereosinophilic syndrome, stroke warming syndrome, transient ischemic attack

## Abstract

**Rationale:**

Few reports of idiopathic hypereosinophilic syndrome exist presenting as ischemic cerebrovascular disease, and the majority are watershed infarction. We report the first case of idiopathic hypereosinophilic syndrome that has clinical features of capsular warning syndrome lasting 6 weeks.

**Patient concerns:**

A 26-year-old man complained of recurrent right limb weakness, accompanying slurred speech, and right facial paresthesia.

**Diagnoses:**

The patient was diagnosed with idiopathic hypereosinophilic syndrome (IHES).

**Interventions:**

Adequate glucocorticoid and anticoagulant treatments were given.

**Outcomes:**

The patient’s motor ability improved, and he was discharged 2 weeks later. Muscle strength in the right-side extremities had fully recovered at a 3-month follow-up after discharge.

**Lessons:**

This case suggests that idiopathic hypereosinophilic syndrome should be considered as a cause of capsular warning syndrome, and the dose of glucocorticoid and the efficacy evaluation index needs to be reevaluated for the treatment of ischemic cerebrovascular disease associated with idiopathic hypereosinophilic syndrome.

## 1. Introduction

Idiopathic hypereosinophilic syndrome (IHES) is a type of hypereosinophilia of unknown origin. It is characterized by sustained peripheral eosinophilia of > 1500 cells/μL of blood with associated end-organ damage.^[1,2]^ Clinical manifestations of hypereosinophilia in the nervous system include cerebral thrombosis, encephalopathy, and peripheral neuropathies. Cases of IHES that present as ischemic cerebrovascular disease are infrequently reported and the majority of reported cases are watershed infarctions. Capsular warning syndrome (CWS) is crescendo episodes of ischemia lasting 24 to 72 hours that are restricted to the region of the internal capsule. This usually causes symptoms affecting the face, arm, and leg,^[3]^ and increases the risk of permanent infarction. Here we describe the first case of IHES that presented with the clinical features of CWS that lasted for 6 weeks. This patient developed cerebral infarction despite receiving adequate glucocorticoid and anticoagulant treatments.

## 2. Case presentation

**Figure 1.**
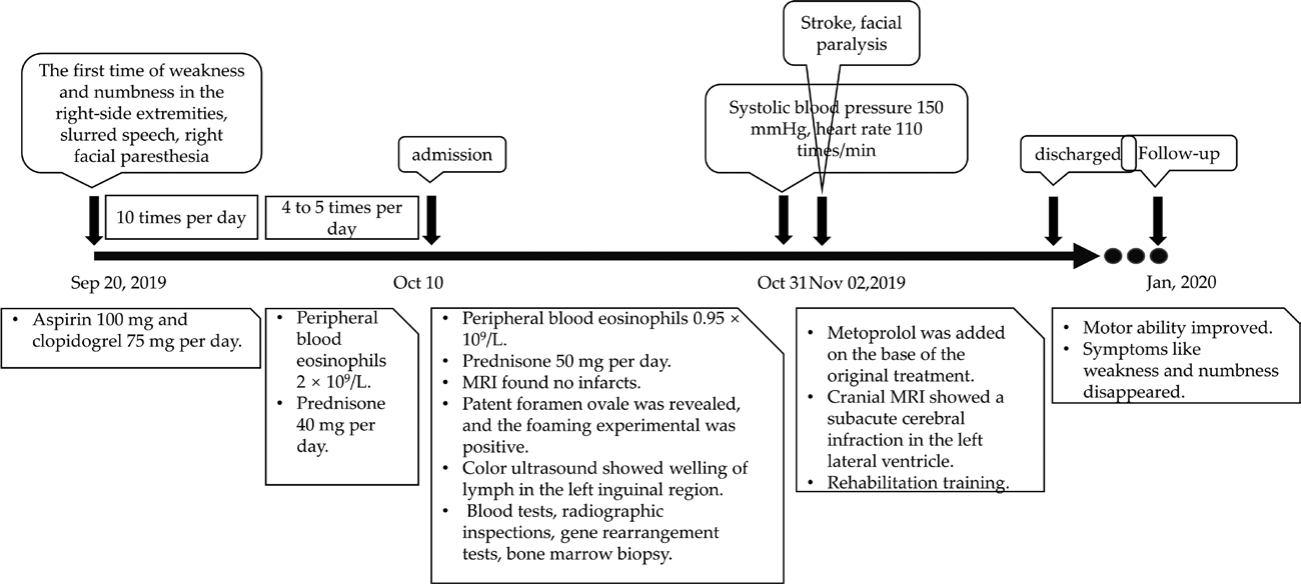
Timeline of intervention and outcomes.

A 26-year-old man was admitted to our hospital on October 10, 2019, due to recurrent right limb weakness. Twenty days prior to admission, the patient developed sudden weakness and numbness in his right upper and lower extremity. The right upper extremity was worse than the right lower extremity. This was accompanied by slurred speech and right facial paresthesia without a clear precipitating factor. These symptoms lasted for several minutes and occurred more than 10 times per day. At a general hospital, the patient received combination therapy consisting of aspirin (100 mg) and clopidogrel (75 mg) per day after a diagnosis of transient ischemic attack (TIA). Without a significant decrease in the number of attacks, he was transferred to a specialty hospital. In that hospital, routine blood tests indicated that peripheral blood eosinophils were elevated (2 × 10^9^/L). Eosinophil-related TIA was diagnosed after reviewing the elevated eosinophils in combination with other symptoms, including rash, variant asthma, and inguinal masses. As a result, the prednisone was increased from the maintenance dose of 5 mg daily for variant asthma to 40 mg per day. Although reduced, episodes of weakness continued to occur 4 to 5 times per day, and the patient was transferred to our hospital (Fig. 1).

**Figure 2.**
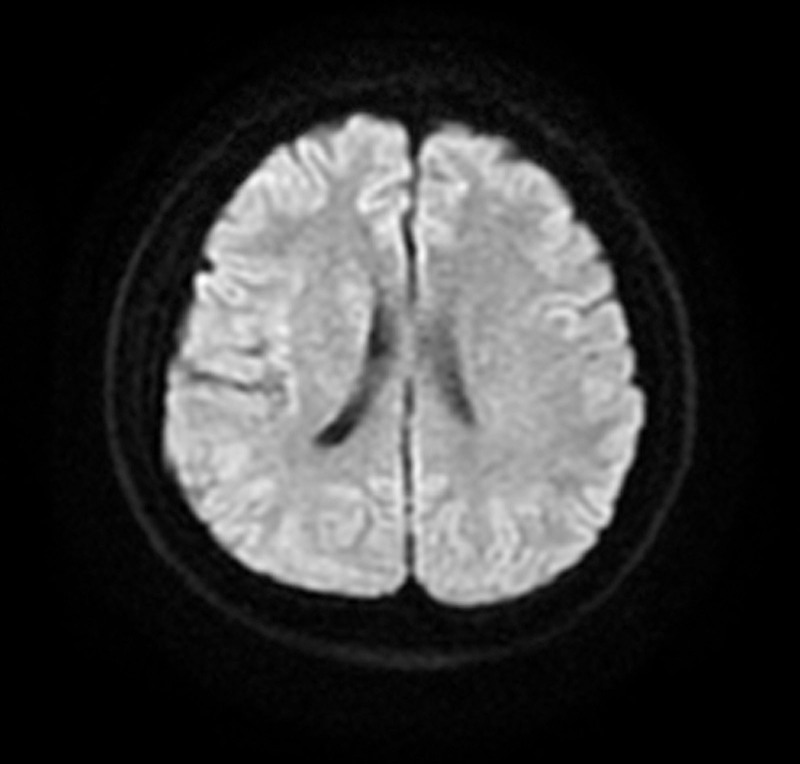
No cerebral infarcts were found using magnetic resonance imaging.

**Figure 3.**
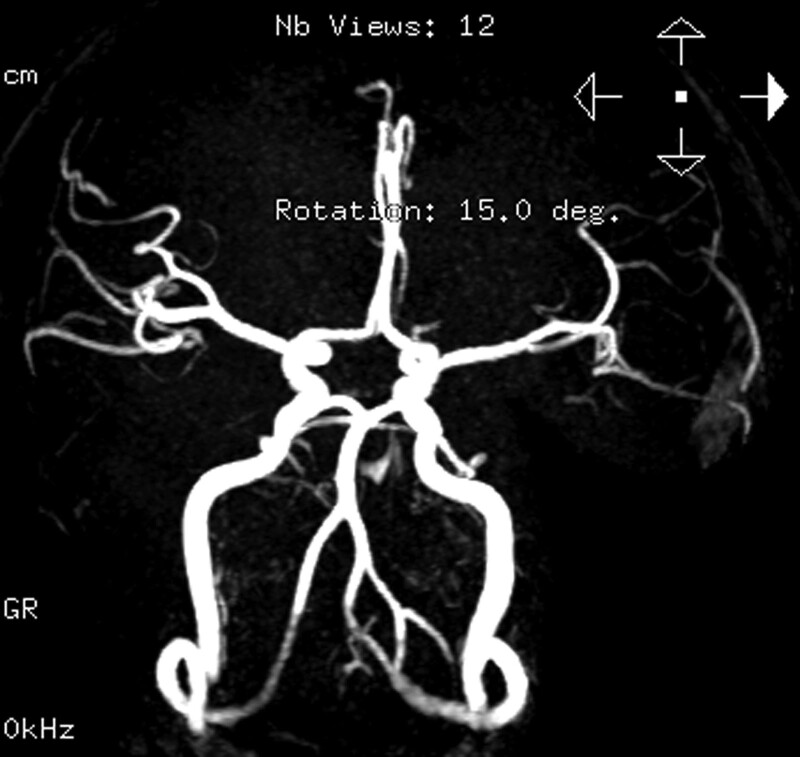
Magnetic resonance angiography of the head revealed local fenestration of the V4 segment in the left vertebral artery and generating branch vessels.

The patient had variant asthma with a maintenance dose of 5 mg prednisone per day, and denied other family history or past histories of drug exposure or travel. At admission, a physical examination did not suggest signs of nervous system involvement, and peripheral blood tests showed that the eosinophil concentration decreased to 0.95 × 10^9^/L. After admission, no cerebral infarcts were found using magnetic resonance imaging (Fig. 2). Magnetic resonance angiography of the head (Fig. 3) revealed local fenestration of the V4 segment in the left vertebral artery and generating branch vessels. The area below the P2 segment of the left posterior cerebral artery was slightly thinner than normal. Ultrasound examination of blood vessels in the neck showed that the bilateral internal carotid and vertebral arteries were normal. Transesophageal echocardiography revealed patent foramen ovale, and the foaming experimental was positive. Arteriovenous Doppler sonography of the extremities was hypoechoic in the bilateral anterior tibial and ulnar arteries with a stenosis rate of 95% to 100%.

Blood tests were negative for anticardiolipin IgG/IgM, anti-β2-glycoprotein IgG/IgM, extractable nuclear antigen antibodies, anti-neutrophil cytoplasmic antibodies, and lupus anticoagulant antibodies. Levels of antinuclear and anti-double-stranded DNA antibodies were < 40 IU/mL and < 100 IU/mL, respectively. Antithrombin III antigen was 33.8 mg/dL, and the activity of protein C and protein S was 119.00% and 99.30%, respectively. Laboratory and radiographic inspection found no evidence of a tumor. Tests for syphilis, human immunodeficiency virus, hepatitis B virus, and hepatitis C virus were negative. No antibodies against parasites or abnormalities in immunity were found.

Morphological analysis of a bone marrow biopsy showed active bone marrow cell proliferation that included granulocyte and erythrocyte hyperplasia, and eosinophilia (the latter accounting for about 20%). Tests for gene rearrangements (*BCRABL1, FIP1L1-PDGFRα, PDGFRβ, FGFR1, PCM1-JACK3,* and *PCM1-JAK2*) were negative and ruled out malignant tumors of the blood that could be associated with an increase in eosinophils. Color ultrasound showed swelling of lymph nodes in the left inguinal region. The morphological and immunohistochemical results indicated that the swelling was due to eosinophilic lymphogranuloma (Kimura disease).

Based on patient history and examinations, we diagnosed TIA caused by IHES and continued the combination of anticoagulation and antiplatelet therapy, but prednisone was increased to 50 mg per day. The patient’s blood eosinophils were controlled within the normal range, and the episodes were reduced to 1 every 4 to 5 days. The majority of these episodes lasted for several minutes. One episode lasted for half an hour and occurred after a contrast agent was injected during computed tomography angiography. After 3 weeks of treatment, the patient was prepared to be discharged. However, his systolic blood pressure and heart rate increased to 150 mm Hg and 110 beats per minute, respectively. Metoprolol was prescribed for the patient.

**Figure 4.**
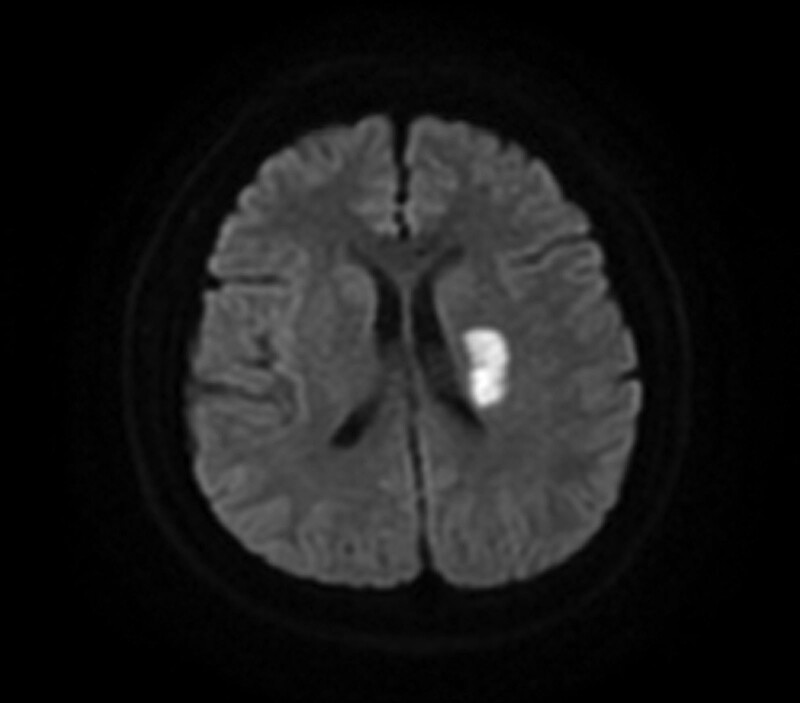
A cranial MRI showed a subacute cerebral infarction in the left lateral ventricle. MRI = magnetic resonance imaging.

Two days later, the patient suffered from weakness of the right side extremities and failed to recover. A physical examination found that the patient was rational but had slurred speech. The right nasolabial groove was shallowed, and the tongue leaned toward the right side. The level of muscle strength on the left side and upper right extremities was 5 and 3, respectively, on the British Medical Research Council muscle strength scale. The bilateral Babinski sign was positive. A cranial magnetic resonance imaging (Fig. 4) showed a subacute cerebral infarction in the left lateral ventricle. Treatment was continued with corresponding rehabilitation training. The patient’s motor ability improved, and he discharged himself from the hospital 2 weeks later. Muscle strength in the right side extremities had fully recovered at a 3 month follow up after discharge.

## 3. Discussion

The clinical symptoms of this patient mainly consisted of repetitive and stereotypical slurred speech and right facial paresthesia accompanied by numbness and weakness of the right side extremities. The patient eventually developed infarction with right hemiplegia and right sided central facial palsy. The left basal ganglia was considered in localization diagnosis, because the patient had symptoms such as right-side central facial paralysis, partial muscle weakness, and hemihypesthesia on the right side and without impacts on awareness, aphasia, and diplopia. It was in the blood supply area of the left middle cerebral artery (MCA). These symptoms and location analysis are consistent with a clinical feature of CWS. No major vascular stenosis was found in the etiological examination. Although the patient had patent foramen ovale, each CWS episode had the same symptoms, which was inconsistent with the characteristics of cardiac emboli involving the anterior and posterior circulation and the left and right drainage basins. Therefore, ischemia of the same vessel area was considered. Consequently, the perforator artery of MCA was considered as the responsible vessel after ruling out major vascular stenosis and cardiac emboli. There was a marked increase in eosinophils and an absence of high-risk factors for arteriosclerosis. Therefore, ischemic cerebrovascular disease caused by IHES was finally considered after ruling out other rare causes of cerebrovascular disease, such as autoimmunity, genetically inherited cancer, cardiogenic embolism, and infection. The patient had no family history or past histories of drug exposure or travel that could explain the presence of IHES or CWS. We considered that it was the first case of CWS caused by IHES.

HES includes 3 main types: idiopathic, myeloproliferative variant, and lymphocytic variant.^[1]^ THES is characterized by tissue and organ damage caused by unexplained peripheral blood or tissue eosinophilia. Almost all systems may be affected by persistent eosinophilia, but the frequency of neurological involvement is low. We searched PubMed for reported clinical cases of cerebral infarctions caused by IHES from 1990 to 2019 (Table 1).^[4–10]^ We found 3 reports of cerebral watershed infarction (CWSI), 2 reports of multiple bilateral infarctions of the brain, 1 report of MCA occlusion, and 1 report of infarction of both anterior and posterior vascular territories. CWSI refers to an ischemic lesion that occurs in the border zone between the territories of 2 major arteries^[11]^ and was thought to be associated with eosinophilic mediated vascular endothelial toxicity in these reports. Active eosinophils release granule proteins that can cause vascular endothelial cell injury and platelet activation, such as major basic protein, eosinophil cationic protein, eosinophil peroxidase, and eosinophil-derived neurotoxin.^[12]^ These proteins can change coagulation and fibrinolysis that may lead to hypercoagulation and microvascular embolization.

**Table 1:** Cases of ischemic stroke caused by idiopathic hypereeosinophilic syndrome.

Age/sex	Medical history	Diagonosis	Area of infarction	Author/year
56/F	Idiopathic hypereosinophilic syndrome (IHES), eosinophilic pneumonia, myocardial infarction, deep venous thrombosis		Punctate infarcts throughout the cerebral and cerebellar hemispheres	Andrea Wasilewski/2019
25/M		Watershed infarction	The arterial border zone	Chen/2017
57/F	Bronchiectasis, hypertension	Watershed infarction	Around ventricles	Chen/2017
56/M		Idiopathic hypereosinophilic syndrome (IHES)	Multiple bilateral cerebral infarcts	Ahn/2010
23/F		MCA occlusion, HES	Middle cerebral artery (MCA) occlusion	Takeuchi/2010
63/F	Sustained eosinophilia, deep venous thrombosis in left lower extremity, patent foramen ovale		Extensive bilateral and diffuse watershed ischemia	Perini/2009
43/M		HES, thromboembolic strokes	Both anterior and posterior vascular territories	Chang/2008
43/M		Cerebral infarction, organic psychosyndrome, IHES		Otto/1993

This patient presented with CWS, which is different from the previously reported IHES-mediated cerebral infarctions noted above. The ischemic attacks in this patient were not in the watershed area but occurred in a perforating artery. CWS was first described by Donnan in 1993 and denotes repetitive, transient cerebral ischemia clustered within a period of short time. CWS is characterized by unilateral motor, sensory, and sensorimotor dysfunction with no cortical signs, combined with a high-risk of internal capsular infarction.^[3]^ Patients are recognized as having CWS when the region of ischemia is classified as being subcortical, and repetitive neurological events occur at presentation. The time frame during which these events occur is usually quite brief, most often within 72 hours or even 7 days. Although the exact pathophysiology of the transient symptoms in CWS is still unknown, there are many possible causes, such as artery-to-artery micro-embolism, hemodynamic events, vasospasms, and other molecular or functional abnormalities.^[3]^ Recently, microscopic polyangiitis has been considered as a possible cause of CWS.^[13]^ It has been postulated that a main mechanism for stroke may be small-vessel, single-penetrator disease that results in low perfusion from hemodynamic changes caused by luminal stenosis.^[3]^

In this case, symptoms of TIA lasted for 6 weeks and had repetitive and stereotypical features, and the cerebral infarction was in the left lateral ventricle, suggesting that the infarction was caused by insufficient irrigation of a single vascular area. Two aspects may be involved in hypoperfusion: stenosis or embolization of the lumen of the perforator artery and hemodynamic changes. The patient had an increase of eosinophils in the peripheral blood, and active eosinophils have complex proinflammatory effects (e.g., direct cytotoxicity, promotion of thrombosis, fibrosis, angiogenesis, tissue remodeling, and platelet and endothelial cell activation^[12]^) that may have caused thrombosis of the bilateral ulnar and anterior tibial arteries. Therefore, it is possible that eosinophilia caused injury to the perforator artery leading to local thrombosis and stenosis of the lumen, or an embolus from another site caused by eosinophilia embolized the perforator artery. Of note, CWS symptoms occurred after the injection of a contrast dye and use of antihypertensive drugs suggesting that lumen size and blood pressure affected the patient’s progress. Injection of a contrast agent may have caused vasospasms and further aggravated lumen stenosis. Antihypertensive treatment may have reduced blood flow resulting in inadequate irrigation in the area surrounding the perforator artery. Therefore, we concluded that local blood flow fluctuations due to stenosis or embolization of the perforator artery caused the recurrent TIAs and the final infarction rather than high coagulation and microvascular embolization found in CWSI. Our conclusion may also explain why CWS episodes lasted for 6 weeks, which is much longer than the normal timeline for CWS.

Although the diagnosis of IHES is straightforward, the treatment is challenging. The first-line drug of choice is prednisone (starting dose of 1 mg/kg/day).^[14]^ Hydroxyurea is an effective agent which may be used in conjunction with corticosteroids or in steroid nonresponders.^[12]^ Other therapeutic options are anti-IL-5 monoclonal antibodies (mepolizumab), interferon-α, cyclophosphamide, and leukapheresis.^[12]^ In addition, IHES-induced cerebral ischemia requires antithrombotic therapy that includes anticoagulation and antiplatelet treatments in addition to immunotherapy. A loading dose of clopidogrel combined with other antithrombotic therapies is reported to be an effective treatment and superior to aspirin alone.^[15]^ Although the number of ischemic attacks in this patient decreased by sufficient prednisone and anticoagulant therapy, his symptoms were not completely controlled and eventually developed into cerebral infarction. Therefore, this treatment strategy should be reevaluated. The level of peripheral blood eosinophils has always been an important indicator for evaluating the efficacy of immunotherapy in patients with hypereosinophilia. Although the peripheral blood eosinophil counts reached the normal range after prednisone treatment, the episodes of transient cerebral ischemia were not completely inhibited which suggests that the level of peripheral blood eosinophils may not fully reflect the effect of IHES on the central nervous system. It is worth exploring whether higher doses of glucocorticoids are required to address central nervous system involvement. Repeated CWS symptoms were aggravated in this patient after the use of a contrast agent and antihypertensive drugs, indicating the existence of hemodynamic changes. Although it is an important strategy to avoid hypoperfusion, we should remain attentive to changes in blood pressure and maintain blood pressure within a reasonable range.

## 4. Conclusion

We report the first case of CWS associated with IHES, which lasted for 6 weeks, and this report suggests that IHES should be considered as a cause of CWS. Despite sufficient glucocorticoid and antithrombotic therapies and normal levels of peripheral blood eosinophils, the IHES evolved into cerebral infarction. These data suggest that the dose of glucocorticoid and the efficacy evaluation index needs to be reevaluated for the treatment of ischemic cerebrovascular disease associated with IHES. In addition, individualized treatment targeting pathogenic mechanisms of cerebral ischemia should be given more attention.

## Acknowledgments

We thank Ruijin Hospital for providing a clinical platform, and the clinical work of all involved doctors and nurses, thank our patient’s permission and support.

## Author contributions

**Investigation:** Kai-Qi Ding, Xuan-Qiang Tu, Yuan-Yue Song.

**Writing – original draft:** Ze-Hua Lai.

**Writing – review & editing:** Li-Li Zeng.
